# WORKFORCE EDUCATION, TECHNOLOGY, AND EVALUATION TO REDUCE HEALTH DISPARITIES IN A PROVIDER SHORTAGE STATE

**DOI:** 10.1093/geroni/igad104.1433

**Published:** 2023-12-21

**Authors:** Ji Yoo, Peter Reed, Laurie Kim

## Abstract

State of Nevada has been a provider shortage area and ranks 50th state of primary care providers per capita in 2022. As a result, Medicare spends highest spending per capita and highest 30-day readmission rate of vulnerable and older adults, i.e., individuals with Alzheimer’s Disease Related Dementias. To respond these challenges, geriatric workforce education program (GWEP) was launched in 2019. Along with interdisciplinary education of healthcare providers, GWEP overarched to an innovative technology, i.e., digital health and machine learning methodology aiming at mitigating bias in healthcare and predicting events, i.e., nursing home placement and designating power of attorney timelines that are useful for planning advance care in primary care. GWEP team evaluates the impact of these education and technology implementation impact on healthcare delivery efficiency, i.e., healthcare cost. in real-world payment system. This is a collaborative symposium between the Community-Engaged Research, Geriatric Education, and Patient/Person Engagement in Research Interest Groups.

